# Validation of the i-STAT system for the analysis of blood gases and acid–base status in juvenile sandbar shark (*Carcharhinus plumbeus*)

**DOI:** 10.1093/conphys/cov002

**Published:** 2015-03-02

**Authors:** T. S. Harter, P. R. Morrison, J. W. Mandelman, J. L. Rummer, A. P. Farrell, R. W. Brill, C. J. Brauner

**Affiliations:** 1 Department of Zoology, University of British Columbia, 6270 University Boulevard, Vancouver, BC, CanadaV6T 1Z4; 2 John H. Prescott Marine Laboratory, New England Aquarium, 1 Central Wharf, Boston, MA 02110, USA; 3 ARC Centre of Excellence for Coral Reef Studies, James Cook University, Townsville, Queensland 4811, Australia; 4 Faculty of Land and Food Systems, University of British Columbia, Main Mall, Vancouver, BC, CanadaV6T 1Z4; 5 National Marine Fisheries Service, Northeast Fisheries Science Center, James J. Howard Marine Sciences Laboratory, 74 Magruder Road, Sandy Hook, Highlands, NJ 07732, USA

**Keywords:** Carbon dioxide tension, elasmobranch, oxygen tension, pH, portable clinical analyser

## Abstract

Accurate measurements of blood gases and acid–base status require an array of sophisticated laboratory equipment that is typically not available during field research; such is the case for many studies on the stress physiology, ecology and conservation of elasmobranch fish species. Consequently, researchers have adopted portable clinical analysers that were developed for the analysis of human blood characteristics, but often without thoroughly validating these systems for their use on fish. The aim of our study was to test the suitability of the i-STAT system, the most commonly used portable clinical analyser in studies on fish, for analysing blood gases and acid–base status in elasmobranchs, over a broad range of conditions and using the sandbar shark (*Carcharhinus plumbeus*) as a model organism. Our results indicate that the i-STAT system can generate useful measurements of whole blood pH, and the use of appropriate correction factors may increase the accuracy of results. The i-STAT system was, however, unable to generate reliable results for measurements of partial pressure of oxygen (*P*O_2_) and the derived parameter of haemoglobin O_2_ saturation. This is probably due to the effect of a closed-system temperature change on *P*O_2_ within the i-STAT cartridge and the fact that the temperature correction algorithms used by i-STAT assume a human temperature dependency of haemoglobin–O_2_ binding; in many ectotherms, this assumption will lead to equivocal i-STAT *P*O_2_ results. The *in vivo* partial pressure of CO_2_ (*P*CO_2_) in resting sandbar sharks is probably below the detection limit for *P*CO_2_ in the i-STAT system, and the measurement of higher *P*CO_2_ tensions was associated with a large measurement error. In agreement with previous work, our results indicate that the i-STAT system can generate useful data on whole blood pH in fishes, but not blood gases.

## Abstract (precis)

We validated the i-STAT system for the analysis of blood parameters in sandbar shark. Results indicate that it is a useful tool for measuring blood pH and could be suitable for operation in most field settings. However, it is not recommended for the assessment of gas tensions in shark blood.

## Introduction

The i-STAT system^®^ (Abbot Point of Care Inc., Princeton, NJ, USA), a portable clinical analyser, is gaining acceptance as a means of blood analysis in biological studies on a variety of fish species (Stoot *et al.*, 2014). This is despite the fact that the i-STAT system was originally developed for the analysis of human blood, hence: (i) samples in the test cartridges are heated to 37°C upon analysis; (ii) results are calculated based on algorithms derived for human blood characteristics (this includes temperature corrections); and (iii) the detection limits of the sensors within the cartridge are optimized for parameter ranges expected in air-breathing mammals. These potential sources of measurement bias require a thorough validation of the i-STAT system when used to analyse blood samples obtained from fishes, taking into account species and sampling conditions. While few studies have validated the i-STAT system for several fish species (seminole killifish, DiMaggio *et al*., 2010; sandbar shark and dusky smooth-hound, Gallagher *et al*., 2010; black and blue rockfish, Harrenstien *et al*., 2005), only one validation study on rainbow trout (*Oncorhynchus mykiss*) has assessed possible interaction effects from a broad range of conditions, by experimentally varying temperature, haematocrit and partial pressure of CO_2_ (*P*CO_2_; Harter *et al*., 2014). Results on rainbow trout indicated that the i-STAT was not appropriate for measuring blood parameters other than blood pH. Consequently, we see no justification for the use of the i-STAT system with rainbow trout in situations where more established analytical techniques are accessible. However, many studies on elasmobranch ecology, physiology and conservation rely on field measurement of blood parameters (e.g. Mandelman and Farrington, 2007a, b; Mandelman and Skomal, 2009; Brooks *et al.*, 2011, 2012; Cicia *et al.*, 2012; Frick *et al.*, 2012; Hyatt *et al.*, 2012), a situation where the i-STAT system may be the only available methodology. Nevertheless, the simple availability of a method cannot justify its implementation; the choice of a suitable method should depend on the quality of the produced data, in terms of precision and accuracy, and on the tolerance of the specific research question to variation in these quality criteria.

Therefore, our aim was to validate the use of the i-STAT system for the analysis of blood gases and acid–base status in elasmobranchs over a range of temperatures, partial pressures of oxygen (*P*O_2_) and *P*CO_2_, using blood from sandbar shark (*Carcharhinus plumbeus*). In addition, we investigated the effects of heating blood samples in a closed system to 37°C, to simulate the temperature changes that occur within i-STAT cartridges during measurements. Our goal was to provide guidelines for an appropriate implementation of the i-STAT system in future studies on elasmobranchs and, based on the results, allow researchers to make an informed decision on whether the i-STAT system is the right tool to answer their specific research questions.

## Materials and methods

This study was carried out as part of a larger project on the effect of temperature on blood–O_2_ binding characteristics in juvenile sandbar shark (P. R. Morrison, T. S. Harter, R. W. Brill and C. J. Brauner, unpublished data). During tonometry, subsamples of blood were also analysed with the i-STAT system, which allowed a direct comparison of the i-STAT system with measurements performed using conventional and proven laboratory techniques.

### Animals and housing

Animal housing and all procedures were approved by the College of William and Mary Animal Care and Use Committee (protocol number: IACUC-2014-04-18-9548-rwbril). Sandbar sharks, *Carcharhinus plumbeus* Nardo 1827 (1.4–8.1 kg), were caught using hook-and-line fishing gear in the tidal lagoon system surrounding the Virginia Institute of Marine Science Eastern Shore Laboratory in Wachapreague, VA, USA. All animals were held for several weeks in a shore-side circular tank (∼8 m in diameter and 2 m deep), supplied with flow-through sea water from the adjacent lagoon (25.7 ± 1.2°C, mean ± SD). Animals were held at a natural photoperiod (June–August), and the tank was shaded with black mesh for protection from direct sunlight. Fish were fed thrice a week with cut pieces of Atlantic menhaden (*Brevoortia tyrannus*), and feeding was suspended 24 h before blood collection.

### Blood collection

After netting a shark out of the holding tank, a blood sample (10–20 ml) was immediately collected into a heparinized syringe (sodium heparin; Sigma 84020) by caudal puncture and placed on ice. Blood samples were subsequently stored at 4°C for several hours to avoid any confounding effects of possible red blood cell swelling immediately after sampling (Brill *et al.*, 2008). Thereafter, 3 ml aliquots of blood were loaded into six Eschweiler tonometers (5 ml total volume), placed in a thermostated water bath and equilibrated with a water-saturated gas mixture (air, CO_2_ and N_2_). Gases were mixed daily, using mass flow controllers (MKS Instruments, Andover, MA, USA) and stored in automobile inner tubes. During mixing of gases, *P*CO_2_ tensions were monitored using an infrared Capnometer HP 47210A (Hewlett-Packard, Böblingen, Germany). All tonometers were flushed with the respective gas mixture for 1 h before loading the blood and allowed to equilibrate for another 1 h before analysis.

### Experimental design

In order to validate the i-STAT system, temperature, *P*O_2_ and *P*CO_2_ were varied with three levels per factor: temperature 15, 20 or 25°C; *P*CO_2_ 0.2 (∼1.52 mmHg), 0.6 (∼4.56 mmHg) or 1.5% (∼11.4 mmHg); and *P*O_2_ 10, 40 or 150 mmHg (balance N_2_). Six replicate samples (*n* = 6) were run for each combination of factors, and the experimental unit was a single tonometer containing blood from a single individual. A total of 171 samples were analysed, using blood from 14 donor fish.

### Sampling and analysis protocols

After equilibration, the six tonometers were sampled sequentially using heparinized, gas-tight Hamilton syringes. A 90 μl subsample was immediately loaded into an i-STAT cartridge; measurements were performed using the VetScan i-STAT 1 System (SN:704583-C; software version JAMS 137a/CLEW A28; Abaxis, Union City, CA, USA) with the i-STAT CG4+ cartridge test. Cartridges were stored in their original packaging at 4°C in the dark and allowed to equilibrate to room temperature over night prior to experiments. All measurements were done within an air-conditioned laboratory and using the temperature correction function of the i-STAT system to account for differences between i-STAT measurements (37°C) and treatment temperatures.

Control measurements of blood parameters were carried out using established laboratory techniques. Haemoglobin (Hb) concentration was measured in triplicate with a Shimadzu UV-1800 spectrophotometer (Kyoto, Japan) using the cyanomethaemoglobin method. Hb concentrations were calculated based on absorption measurements at 540 nm and using an extinction coefficient of 11. Whole blood pH and *P*O_2_ measurements were performed using two Radiometer BMS 3 Mk2 systems and Radiometer acid–base analysers PHM73 (Copenhagen, Denmark). One unit was thermostated at the respective treatment temperature (15, 20 or 25°C) and the other at 37°C, to simulate the closed-system temperature change that occurs to blood samples analysed within an i-STAT cartridge. Whole blood total O_2_ content (TO_2_) was measured according to Tucker (1967). Hb–O_2_ saturation (sO_2_) was calculated from TO_2_ after subtracting physically dissolved O_2_ according to Boutilier *et al*. (1984) and dividing by the theoretical maximal carrying capacity of the rinsed red blood cells based upon the tetrameric Hb concentration obtained spectrophotometrically.

### Data analysis

All data were analysed with RStudio 0.98.1049 (RStudio Inc., Boston, MA, USA). The i-STAT values were compared with control measurements by regression analysis using the raw data. The measurement errors for the i-STAT values relative to control measurements were calculated as follows: δ = (i-STAT − control)/control × 100. The δ data were then compared with control measurements either by regression analysis or by fitting a non-linear model to the data. Linear, logarithmic and exponential models were compared using the Akaike information criterion (AIC), and the model with the best fit (i.e. with the lowest AIC value) was used as representative for the data. Normality of distribution was tested with the Shapiro–Wilk test (*P* < 0.05), and homogeneity of variances was tested with the Levene's test (*P* < 0.05). The effects of temperature, *P*O_2_ and *P*CO_2_ on δ were tested on the squared values of δ (i.e. all values were positive). In most cases, this transformation led to a significant deviation of the distribution from normality, which could not be remediated by data transformation. Therefore, the effects of temperature, *P*O_2_ and *P*CO_2_ on δ were tested with the Kruskal–Wallis rank sum test (*P* < 0.05, *n* = 171; minus missing values as indicated in Table 2) and the Wilcoxon rank sum test (*P* < 0.05, *n* = 108; minus missing values as indicated in Table 2) for the effect of *P*CO_2_ on δ*P*CO_2_. All data are presented as means ± SEM.

## Results

### pH

**Figure 1:**
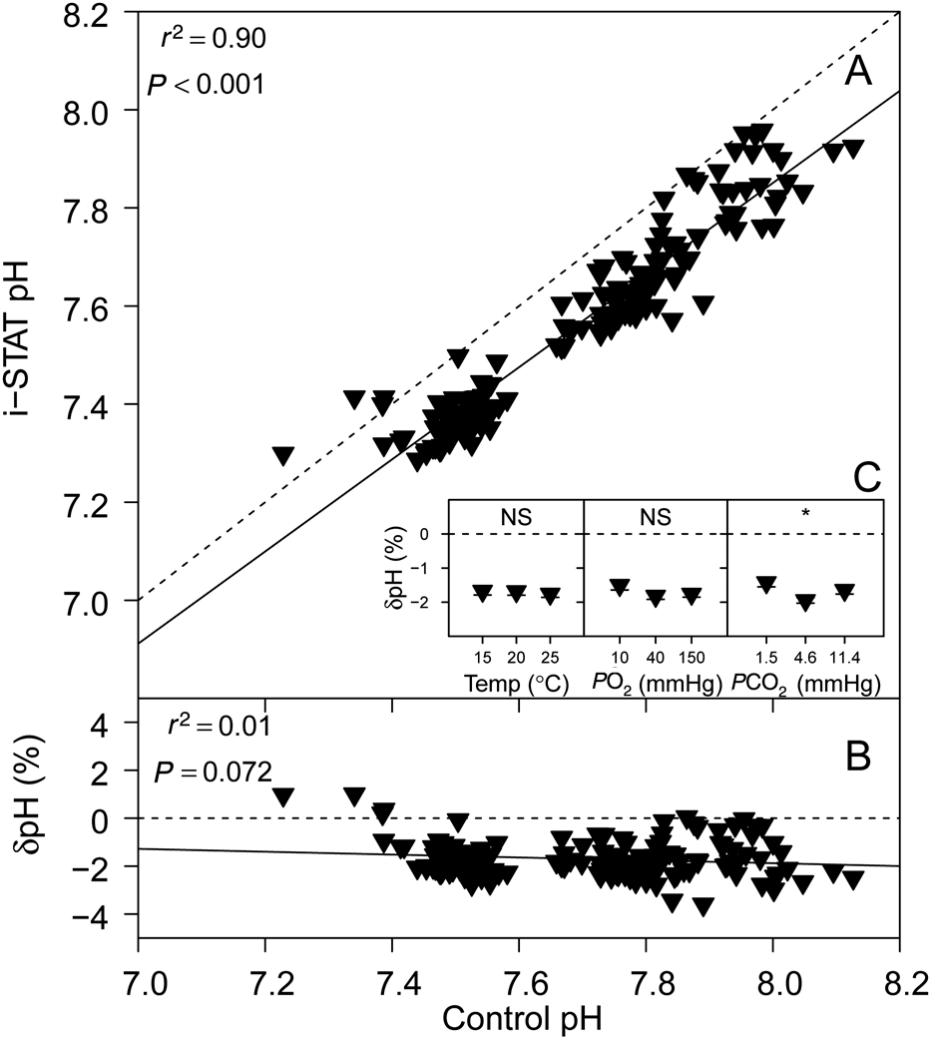
(**A**) Sandbar shark whole blood pH measured with the i-STAT system (temperature-corrected values) vs. pH measured using a thermostated electrode (control). (**B**) The relative error of i-STAT pH measurements, δpH [expressed as %; calculated as (i-STAT pH − control pH)/control pH × 100], vs. control pH. Continuous lines represent the fitted linear models (see Table 1 for parameter estimates) and dashed lines represent the lines of identity. (**C**) Effects of temperature (in °C), partial pressures of oxygen (*P*O_2_) and carbon dioxide (*P*CO_2_; in mmHg) on δpH. Significant effects within treatments are indicated as ‘*’ at the *P* < 0.05 level or NS for non-significant. Data are means ± SEM, and statistical analysis was performed on the squared δpH values.

Regression analysis yielded a highly significant linear relationship between pH measurements performed with the i-STAT system in comparison to control pH measurements using a thermostated electrode (Fig 1A). The measurement error of i-STAT pH measurements, δpH (%), relative to control pH measurements is shown in Fig. 1B. No significant relationship between δpH and control pH was detected (parameter estimates are presented in Table 1). *P*CO_2_ had a significant effect on δpH (*P* = 0.004), but no significant effects were detected for temperature (*P* = 0.704) or *P*O_2_ (*P* = 0.277; Fig. 1C).

**Table 1:** Parameter estimates (means ± SEM), *r*^2^ and *P*-values for the relationships between i-STAT system vs. control measurements, i-STAT measurement errors, δ(*x*) (as %) vs. control measurements (*n* = 171) and the effect of a closed-system temperature increase on pH and *P*O_2_ (*n* = 54)

Measurement	*a*	*b*	*c*	*r*^2^	*P*-value
pH	0.338 ± 0.197	0.939 ± 0.026		0.899	<0.001
δpH	2.917 ± 2.556	−0.599 ± 0.331		0.015	0.072
*P*O_2_	7.079 ± 2.272	0.666 ± 0.027		0.791	<0.001
δ*P*O_2_	8.972 ± 3.358	−0.283 ± 0.040		0.235	<0.001
*P*CO_2_	−0.291 ± 0.120	0.812 ± 0.014		0.969	<0.001
δ*P*CO_2_	−27.718 ± 1.798	0.560 ± 0.209		0.054	0.009
sO_2_	−148.614 ± 8.725	0.966 ± 0.003	−106.295 ± 1.880		
δsO_2_	105.151 ± 4.437	−1.030 ± 0.061		0.731	<0.001
Closed-system pH					
15°C	3.008 ± 0.821	0.564 ± 0.110		0.599	<0.001
20°C	2.845 ± 0.928	0.587 ± 0.123		0.575	<0.001
25°C	3.934 ± 1.176	0.448 ± 0.157		0.295	0.012
Closed-system *P*O_2_					
15°C	−271.305 ± 13.020	0.975 ± 0.003	−249.500 ± 8.364		
20°C	−232.953 ± 10.134	0.981 ± 0.003	−220.472 ± 9.990		
25°C	−234.581 ± 14.486	0.988 ± 0.002	−226.707 ± 18.387		

Abbreviations: *P*CO_2_, partial pressure of carbon dioxide; *P*O_2_, partial pressure of oxygen; sO_2_, haemoglobin O_2_ saturation.

Linear relationships according to: i-STAT(*x*) = *a* + *b* × control(*x*); and δ(*x*) = *a* + *b* × control(*x*).

Exponential relationships according to: i-STAT(*x*) = *a* × *b*^control(*x*)^ − *c*.

Closed-system:

Linear relationships according to: 37°C(*x*) = *a* + *b* × treatment temperature(*x*).

Exponential relationships according to: 37°C(*x*) = *a* × *b*^treatment temperature(*x*)^ − *c*.

All parameter estimates in non-linear models were statistically significant ('*t*-test, *P* < 0.001).

### Partial pressure of O_2_

**Figure 2:**
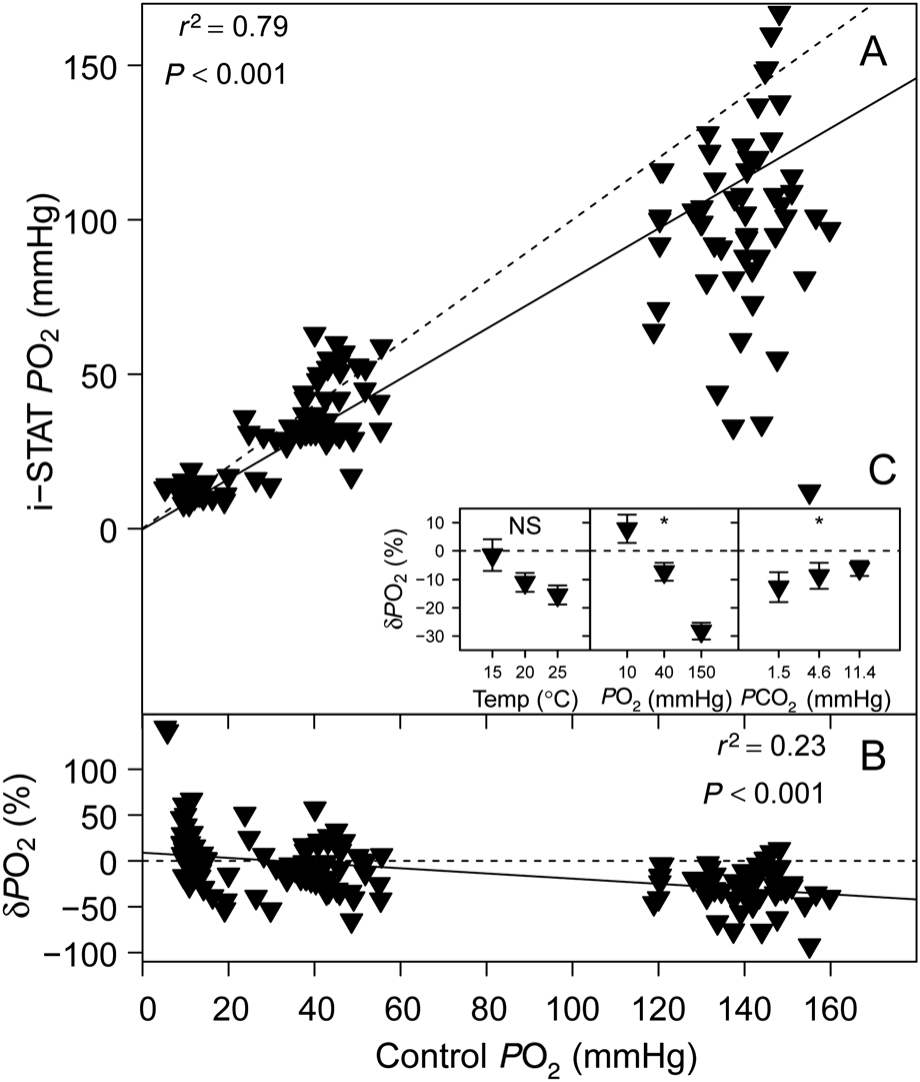
(**A**) Sandbar shark whole blood *P*O_2_ measured with the i-STAT system (temperature-corrected values) vs. *P*O_2_ measured with a thermostated electrode (control). (**B**) The relative error of i-STAT *P*O_2_ measurements, δ*P*O_2_ vs. control *P*O_2_. (**C**) Effects of temperature, *P*O_2_ and *P*CO_2_ on δ*P*O_2_. See legend to Fig. 1 for further information.

A highly significant linear relationship was detected between i-STAT *P*O_2_ and control *P*O_2_ (Fig. 2A) and between i-STAT *P*O_2_ and δ*P*O_2_ (Fig. 2B). Both *P*O_2_ (*P* < 0.001) and *P*CO_2_ (*P* = 0.014) had a significant effect on δ*P*O_2_, but there was no significant effect of temperature on δ*P*O_2_ (*P* = 0.062; Fig. 2C).

### Partial pressure of CO_2_

**Figure 3:**
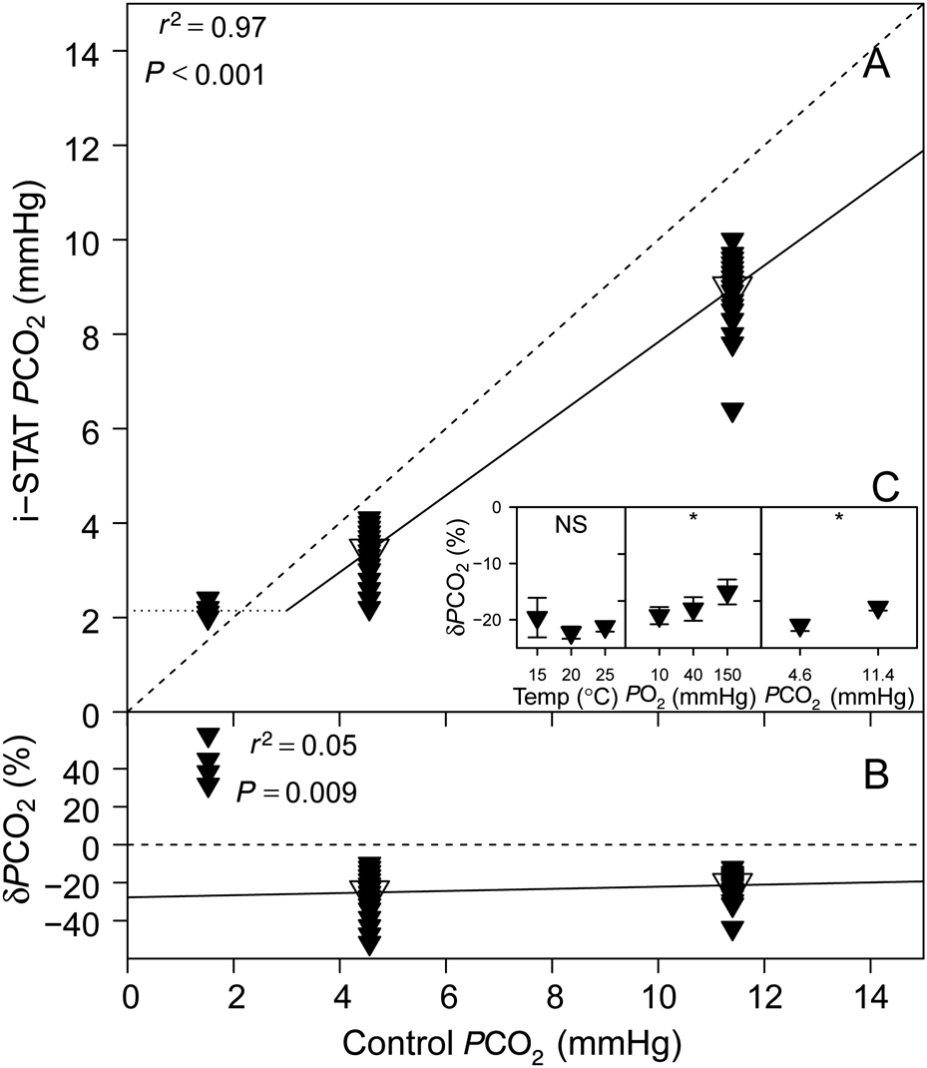
(**A**) Sandbar shark whole blood *P*CO_2_ measured with the i-STAT system (temperature-corrected values) vs. set *P*CO_2_ in the tonometers (control). Mean values are indicated by the larger, open symbols. (**B**) The relative error of i-STAT *P*CO_2_ measurements, δ*P*CO_2_ vs. control *P*CO_2_. The dotted line is the lowest reportable *P*CO_2_ tension by the i-STAT system at 20°C. (**C**) Effects of temperature, *P*O_2_ and *P*CO_2_ on δ*P*CO_2_. See legend to Fig. 1 for further information.

There was a highly significant linear relationship between i-STAT *P*CO_2_ and control *P*CO_2_ (Fig. 3A), and regression analysis detected a significant linear relationship between δ*P*CO_2_ and control *P*CO_2_ (Fig. 3B). Both *P*O_2_ (*P* = 0.005) and *P*CO_2_ (*P* < 0.001) had significant effects on δ*P*CO_2_, but there was no significant effect of temperature on δ*P*CO_2_ (*P* = 0.427; Fig. 3C).

### Haemogloblin saturation

**Figure 4:**
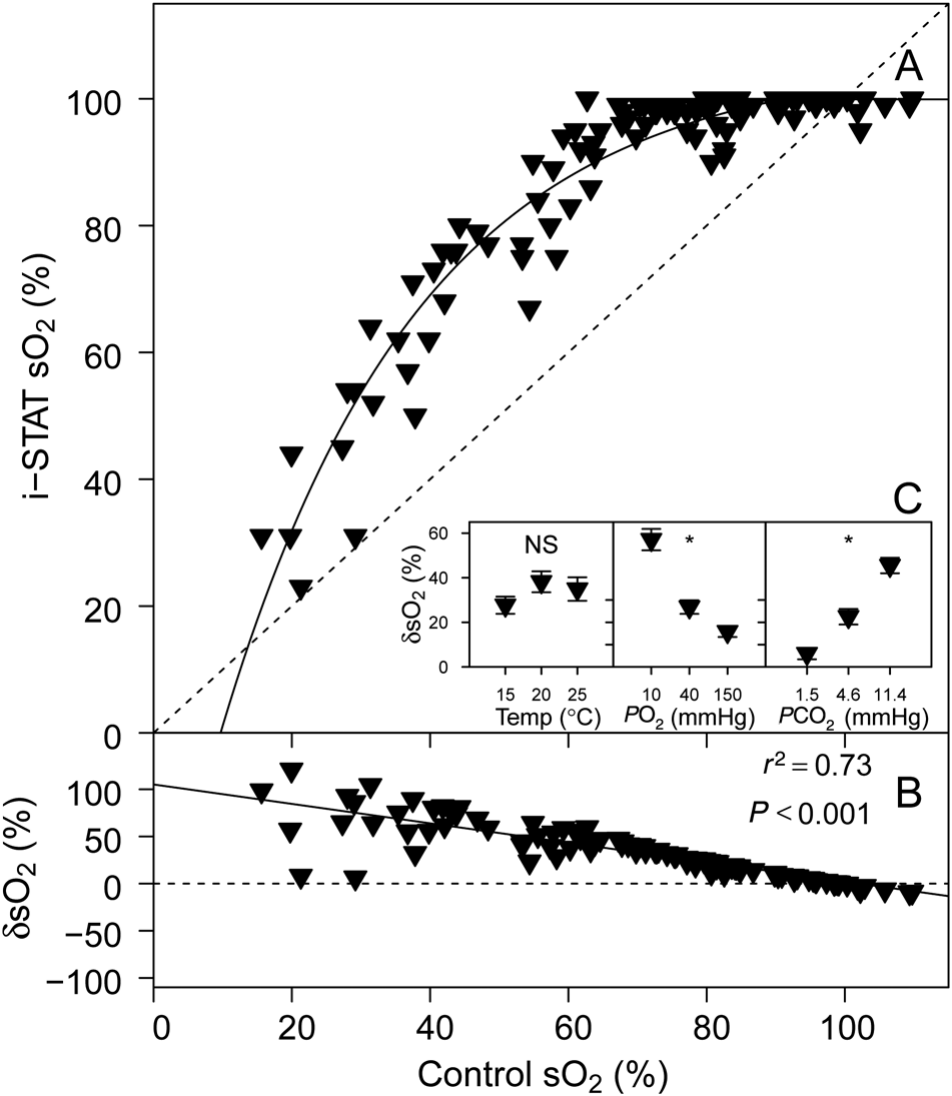
(**A**) Sandbar shark haemoglobin O_2_ saturation (sO_2_) measured with the i-STAT system vs. control sO_2_ measured according to Tucker (1967). (**B**) The relative error of i-STAT sO_2_ measurements, δsO_2_ vs. control sO_2_. (**C**) Effects of temperature, *P*O_2_ and *P*CO_2_ on δsO_2_. See legend to Fig. 1 for further information.

The relationship between i-STAT sO_2_ and control sO_2_ was best described by an exponential model (AIC = 669) rather than a linear (AIC = 780) or logarithmic model (AIC = 706; Fig. 4A). There was also a highly significant relationship between i-STAT δsO_2_ and control sO_2_ (Fig. 4B). The factors *P*O_2_ (*P* < 0.001) and *P*CO_2_ (*P* < 0.001) had significant effects on δsO_2_, but there was no significant effect of temperature on δsO_2_ (*P* = 0.197; Fig. 4C).

### Closed-system temperature effects

**Figure 5:**
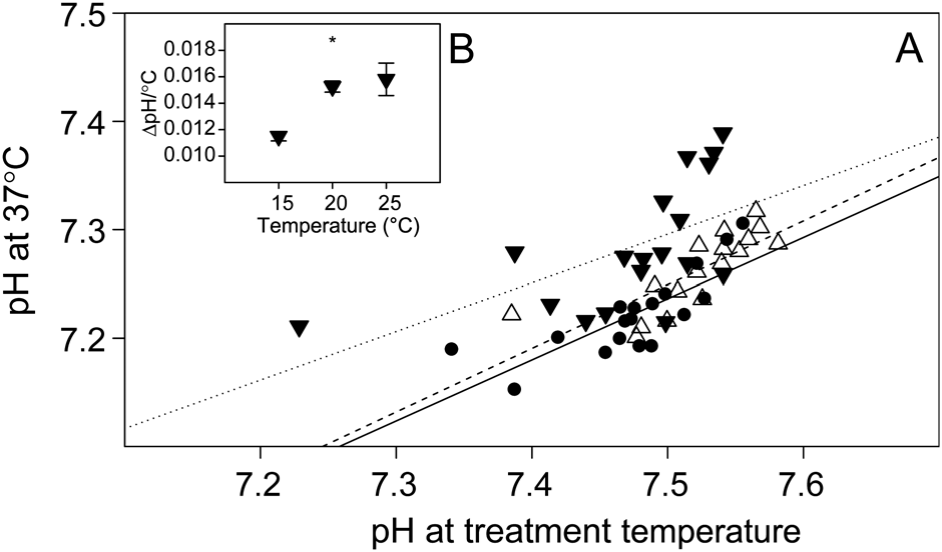
(**A**) Effect of a closed-system temperature increase (from treatment temperature to 37°C) on pH of sandbar shark whole blood, equilibrated in tonometers at 15 (filled circles), 20 (open trianges) or 25°C (inverted filled triangles). Measurements were performed simultaneously with two Radiometer BMS systems, one maintained at the respective treatment temperature and the other at 37°C. The continuous (15°C), dashed (20°C) and dotted (25°C) lines represent the fitted linear models, and the best model was determined by comparing the Akaike information criteria of linear, exponential and logarithmic models. (**B**) ΔpH per degree Celsius (means ± SEM) during a closed-system temperature increase from treatment temperature to 37°C. Significant effects of temperature are indicated by ‘*’ at the *P* < 0.05 level.

**Figure 6:**
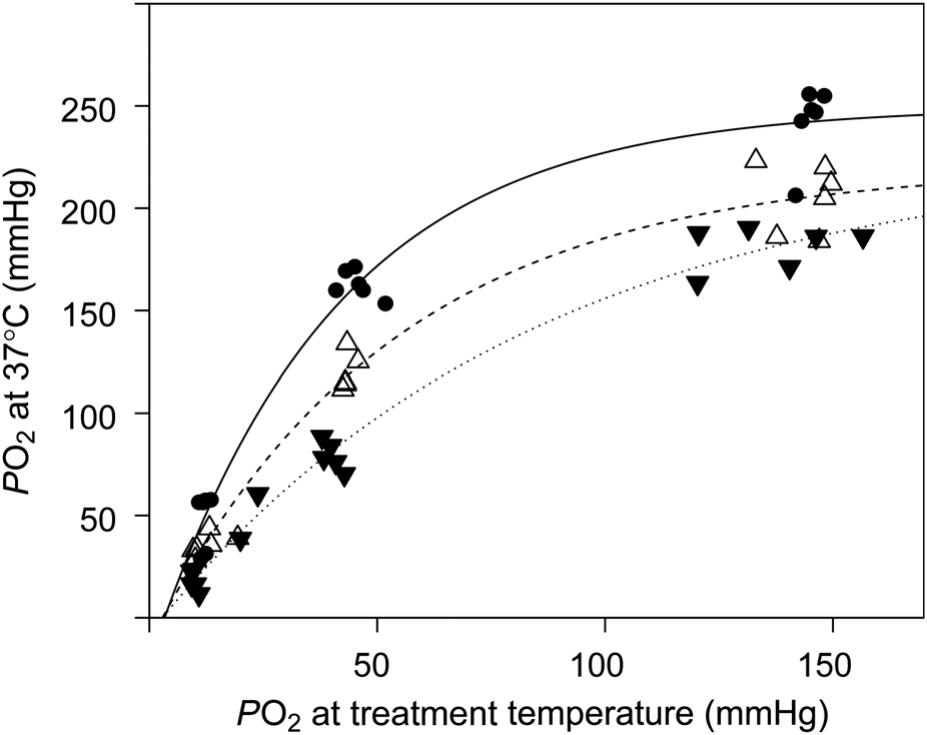
Effect of a closed-system temperature increase (from treatment temperature to 37°C) on *P*O_2_ (in mmHg) of sandbar shark whole blood, equilibrated in tonometers at 15 (filled circles), 20 (open trianges) or 25°C (inverted filled triangles). See legend to Fig. 5 for further information.

There was a significant linear relationship between blood pH measured at treatment temperature (15, 20 or 25°C) and pH measured at 37°C after closed-system heating (Fig. 5A). No significant differences (*P* > 0.05) were detected between the slopes of the linear relationships. There was, however, a significant effect (*P* < 0.001) of treatment temperature on ΔpH per degree Celsius (Fig. 5B). The relationships between *P*O_2_ measured at treatment temperature and at 37°C were best described by exponential models (Fig. 6).

### Failed measurements

Failed measurements (i.e. where the i-STAT system did not give complete results) are summarized in Table 2. This table excludes those measurements that failed due to human error (e.g. loading of blood into the cartridge) or due to defective cartridges. Out of 171 measurements, 59 cartridges (34.5%) failed to give complete results. While pH was generally measured reliably, blood gases (especially *P*CO_2_) were not. In all cases, missing *P*O_2_ values were flagged with ‘***’, indicating that results were not reportable based on the internal quality control rejection criteria of the i-STAT system. In the case of failed *P*CO_2_ readings, four out of 58 (6.9%) were flagged by ‘***’, while the remainder were below the reportable range for *P*CO_2_ (i.e. reported as <5 mmHg; i-STAT Procedure manual, 2014).

**Table 2:** Missing values as reported by the i-STAT system grouped by treatment (*n* = 171)

Temperature (°C)	ipH (%)	i*P*O_2_ (%)	i*P*CO_2_ (%)
15	5.0	1.7	30.0
20	1.4	1.9	35.2
25	14.0	1.8	36.8
*P*O_2_ (mmHg)			
10	17.5	3.5	35.1
40	5.3	0.0	33.3
150	3.5	1.8	33.3
*P*CO_2_ (mmHg)			
1.52	24.6	3.5	93.0
4.56	0.0	0.0	6.7
11.40	1.9	1.9	1.9

Certain combinations of experimental factors were more likely to cause faulty measurements. At the lowest *P*CO_2_ tested (1.52 mmHg), the i-STAT system was unable to report *P*CO_2_ in 93% of cases. This is not surprising, because the detection limit for *P*CO_2_ in the i-STAT system is 5 mmHg, as specified by the manufacturer. Interestingly, at 1.52 mmHg *P*CO_2_, *P*O_2_ measurements failed in 25% of the samples, even though *P*O_2_ was always within detection limits. Also, low *P*O_2_ tensions (10 mmHg) and high temperatures (25°C) seemed to increase the occurrence of failed pH measurements with the i-STAT system in sandbar shark whole blood.

## Discussion

Our results indicate that the i-STAT system is an appropriate tool for the measurement of whole blood pH in the sandbar shark. However, *P*O_2_ and *P*CO_2_ could not be measured accurately, and we cannot recommend the use of this instrument to assess blood gas tensions and any derived parameters in sandbar sharks under the tested range of conditions.

In agreement with previous work (Harter *et al.*, 2014), our results indicate that i-STAT measurements of sandbar shark whole blood pH were accurate, and on average only 1.65 ± 0.07% lower than control pH measurements with a thermostated electrode. This is in line with previous studies on fish, which found similar measurement errors for pH (δpH), as follows: −3.8 for sandbar shark and −4.4% for dusky smooth-hound (calculated from the data presented by Gallagher *et al.*, 2010); −5% for black rockfish (Harrenstien *et al.*, 2005); and 2% for rainbow trout (Harter *et al.*, 2014). Furthermore, we detected no significant effects of temperature or *P*O_2_ on i-STAT δpH, indicating that measurements will remain accurate even if these conditions vary. The significant effect of *P*CO_2_ on δpH indicates that changes in blood *P*CO_2_ may affect the accuracy of i-STAT pH measurements. Even so, δpH was within <4% of control measurements for all pH measurements performed with the i-STAT system (*n* = 156).

We observed a high individual variation among i-STAT pH measurements, typically ∼0.2 pH units, which is perhaps a result of single-point measurements, whereas the BMS system allows the user to make continuous readings to reduce within-sample variation. Whether i-STAT pH measurements are suitable to answer certain research questions will depend on the tolerance for variation within the specific experiment; in any case, the expected variation needs to be considered in the experimental design (e.g. by increasing the number of replicates if greater accuracy of means is required).

Given that the i-STAT system was developed for the analysis of human blood, samples in i-STAT cartridges are heated to 37°C; these cartridges can be considered a closed system, because exchanges of protons, proton equivalents or O_2_ with the environment are negligible. A closed-system temperature change, however, can have marked effects on whole blood pH and *P*O_2_, a phenomenon that we examined separately in order to further assess the suitability of the i-STAT system for the measurement of these blood parameters in ectothermic fish. In line with the theoretical considerations described by Malte *et al.* (2014) underlying a closed-system temperature change, we found a decrease in blood pH when blood was heated to 37°C (Fig. 5A). As expected, the magnitude of this pH change was dependent on the temperature gradient (i.e. heating blood from 15 to 37°C had a larger effect on pH than heating from 25 to 37°C), but in a non-linear manner for sandbar shark blood (Fig. 5B). The i-STAT system uses the pH-temperature dependency for human blood (−0.0147 ΔpH/°C, Rosenthal, 1948) to correct pH values from 37°C to treatment temperature (i-STAT Technical Bulletin, 2013b). The fact that the i-STAT system underestimated control pH by ∼0.1 pH units may be indicative that, over the tested range of pH values and temperatures, the average pH temperature dependency of sandbar shark whole blood was greater than that used for human blood. Despite this bias, the temperature correction algorithm used by the i-STAT (see parameter estimates in Table 1) yielded better results compared with the temperature correction of i-STAT raw values proposed by Mandelman and Skomal (2009), although differences were small (0.835 ± 0.023 × control pH + 1.087 ± 0.176, *P* < 0.001, *r*^2^ = 0.898). Therefore, the parameter estimates provided in Table 1 and our results of closed-system heating on blood pH (i.e. the non-linearity of ΔpH per degree Celsius over the tested temperature range) can be used to correct i-STAT pH measurements and thereby increase their accuracy. It needs to be emphasized, however, that the presented linear relationships are likely to be species specific and are limited to the range of test conditions that have been examined (for comparison see Gallagher *et al.*, 2010).

In contrast to our findings on pH, *P*O_2_ measurements on sandbar shark blood with the i-STAT were unreliable. The fitted linear relationship between i-STAT and control *P*O_2_ measurements (Fig. 2A) indicates that, at least at lower *P*O_2_, the i-STAT *P*O_2_ values were consistent with control measurements, but at higher *P*O_2_ the variability of i-STAT *P*O_2_ measurements increased considerably. However, the calculated measurement errors (Fig. 2B) indicate that at low *P*O_2_, the accuracy of i-STAT *P*O_2_ measurements was also poor, varying between +50 and −50%. At 150 mmHg *P*O_2_, the i-STAT system on average underestimated control *P*O_2_ by −28%, which is reflected in a significant effect of *P*O_2_ on δ*P*O_2_ (Fig. 2C). The high variability of i-STAT *P*O_2_ measurements at 150 mmHg *P*O_2_ is not only indicative of an unreliable measurement, but also prohibits the use of linear equations to correct i-STAT results, because the assumption of homoscedasticity in linear regression analysis was violated. In addition, *P*CO_2_ had a significant effect on the i-STAT *P*O_2_ measurement error and therefore changes in *P*CO_2_ will affect the accuracy of i-STAT *P*O_2_ measurements. Gallagher *et al.* (2010), who previously validated the i-STAT for blood gases in sandbar shark with the same BMS system, found a significant linear relationship between i-STAT *P*O_2_ and control measurements, without the large variability that we observed. Underlying this difference between the two studies is undoubtedly the broader range of test conditions that were examined here. Gallagher *et al.* (2010) analysed sandbar shark blood at one temperature (25°C), while we tested *P*O_2_ at three temperatures (15, 20 and 25°C), discovering no significant effect of temperature on δ*P*O_2_ for the temperature-corrected i-STAT values (*P* = 0.062; Fig. 2C). There was, however, a highly significant (*P* < 0.001) temperature effect on the δ*P*O_2_ of the raw i-STAT data (i.e. without temperature correction; data not shown).

In agreement with the results of Malte *et al.* (2014), the closed-system temperature increase that occurs in an i-STAT cartridge resulted in a dramatic increase in *P*O_2_ of sandbar shark whole blood, nearly doubling the initial values (Fig. 6). It is these high *P*O_2_ tensions that will be analysed by the i-STAT and represent the basis for subsequent temperature correction of the results. According to the equations presented by Malte *et al.* (2014), the measurement error for *P*O_2_ after temperature correction will increase linearly with increasing initial *P*O_2_ and exponentially with increasing temperature gradient. These predictions are entirely in line with our results and may help to explain the increasing variability of i-STAT *P*O_2_ measurements with increasing initial *P*O_2_ (Fig. 2A). Also, measurement errors are likely to be augmented by the differences in temperature dependency of Hb–O_2_ binding between shark and human blood (P. R. Morrison, T. S. Harter, R. W. Brill and C. J. Brauner, unpublished data). The complexity of these interactions is further aggravated by the fact that pH and *P*CO_2_ will also change during closed-system heating, and both of these factors typically alter Hb–O_2_ binding properties in sandbar shark blood (Brill *et al.*, 2008). Collectively, these considerations raise general concerns about the accuracy of blood *P*O_2_ measurements for ectothermic species using any portable clinical analyser that operates at 37°C. As indicated by our results, the closed-system temperature effects on *P*O_2_ can be significant and may not be easily corrected over a wide range of species and conditions. Therefore, due to the high variability of i-STAT *P*O_2_ measurements and a significant effect of *P*CO_2_ on δ*P*O_2_, we do not consider the i-STAT system an appropriate tool for measuring *P*O_2_ in sandbar shark whole blood, or likely other fish species.

In the i-STAT system, sO_2_ is calculated from the measured values of *P*O_2_ and pH (for a summary of the methods used by the i-STAT system refer to Table 2 of Harter *et al*., 2014). Control sO_2_ values were varied experimentally from 20 to 100% (by changing tonometer *P*O_2_ and *P*CO_2_), and our results indicate that, over this range, the i-STAT system overestimated sO_2_. In a previous validation study on trout (Harter *et al.*, 2014), the i-STAT system consistently reported 100% sO_2_ over the entire range of control sO_2_ values. For sandbar shark blood, there was some response of i-STAT sO_2_ to the planned contrasts in sO_2_ (which may reflect the absence of a strong Bohr–Root effect in sharks compared with trout; Berenbrink *et al*., 2005). However, control sO_2_ values above ∼60% were reported as full Hb saturation (i.e. 100%). In normoxic resting fish, sO_2_ values typically range from 100% in the arterial system to 50% sO_2_ in the venous system (Brauner and Randall, 1998); over this range, the i-STAT was unable to detect relative differences by largely reporting 100% sO_2_. Venous sO_2_ in fish will decrease below 50% during exercise and hypoxia (Brauner and Randall, 1998; Brauner *et al*., 2000), while arterial sO_2_ can be lower than 50% during severe hypoxia (Brauner *et al.*, 2001). However, i-STAT measurements of sO_2_ over this range were associated with a measurement error ranging from 50 to 100%. Furthermore, the highly significant effects of *P*O_2_ and *P*CO_2_ on δsO_2_ indicate that changes in these factors will affect the accuracy of sO_2_ measurements with the i-STAT system. Sandbar shark have an exceptionally low *P*_50_ (the partial pressure of O_2_ at which Hb is 50% saturated) of <5 mmHg at 15°C (P. R. Morrison, T. S. Harter, R. W. Brill and C. J. Brauner, unpublished data), which indicates that their Hb will most likely be nearly fully saturated with O_2_ in a broad range of environmental conditions. Consequently, it seems that the i-STAT system cannot generate accurate sO_2_ readings on sandbar shark blood, and it seems unlikely that it would be able to detect relative differences in sO_2_ occurring under most conditions *in vivo*.

The three *P*CO_2_ tensions (1.52, 4.56 and 11.40 mmHg) that we used broadly cover the *P*CO_2_ tensions expected in sandbar shark blood *in vivo*, from resting *P*CO_2_ to extreme hypercapnia during exhaustive exercise (Piiper *et al.*, 1972; Holeton and Heisler, 1983; Richards *et al.*, 2003). Interestingly, studies that have assessed *P*CO_2_ tensions in exhaustively exercised sharks using conventional *P*CO_2_ electrodes found no significant increase in arterial *P*CO_2_ (Richards *et al.*, 2003) or only a moderate increase (5 mmHg, Holeton and Heisler, 1983; 3 mmHg, Piiper *et al.*, 1972), whereas i-STAT measurements generally report higher *P*CO_2_ values (e.g. Mandelman and Skomal, 2009; Hyatt *et al.*, 2012; Naples *et al.*, 2012). We decided to validate the i-STAT system for the range of *P*CO_2_ values that are commonly reported in literature, including those values generated with the i-STAT system itself. Whether *P*CO_2_ tensions as high as 11.4 mmHg are representative of *in vivo* conditions in sharks was not the subject of this investigation, and to our knowledge this remains to be thoroughly assessed.

The lower detection limit of the *P*CO_2_ electrode used in i-STAT cartridges is 5 mmHg at 37°C. However, the i-STAT will report temperature-corrected *P*CO_2_ values below this detection limit according to: min*P*CO_2_ = 5 × 10^0.019^(*T*−37), where min*P*CO_2_ (in mmHg) is the detection limit of the i-STAT for *P*CO_2_ and *T* is the treatment temperature (in °C; i-STAT Technical Bulletin, 2013a). For our test temperatures, the theoretical detection limit of the i-STAT for *P*CO_2_ is 1.9, 2.4 and 3.0 mmHg at 15, 20 and 25°C, respectively (see dotted line in Fig. 3A). Consequently, our lowest test *P*CO_2_ tension (1.52 mmHg) was below the detection limit at every treatment temperature, and the i-STAT system reported values for only 7% of the 57 measurements (Table 2). We therefore excluded the lowest *P*CO_2_ from statistical analysis, and the linear relationships were based on the *P*CO_2_ values of 4.56 and 11.40 mmHg only. Unlike the situation for rainbow trout (where *P*CO_2_ was overestimated by the i-STAT system), it appears that in sandbar shark blood the i-STAT underestimated *P*CO_2_ by ∼20%. Yet, in both studies, δ*P*CO_2_ decreased at higher *P*CO_2_ tensions and would be <1% at a *P*CO_2_ of 19 mmHg in rainbow trout and 50 mmHg in sandbar shark. The δ*P*CO_2_ in rainbow trout scaled exponentially with control *P*CO_2_ (Harter *et al.*, 2014), but the same could not be confirmed for sandbar shark blood after excluding our lowest *P*CO_2_ tension. The different outcomes of i-STAT *P*CO_2_ measurements in trout and sandbar sharks may be the result of differences in *P*O_2_. Harter *et al.* (2014) nominally set *P*O_2_ to 46 mmHg, whereas we varied *P*O_2_ over three levels and found a significant effect of *P*O_2_ on δ*P*CO_2_. Therefore, changes in *P*O_2_ will affect the accuracy of i-STAT *P*CO_2_ measurements, and overall, higher values of *P*O_2_ and *P*CO_2_ will yield more accurate i-STAT *P*CO_2_ readings. We cannot recommend the use of the i-STAT system for measuring *P*CO_2_ in sandbar sharks because: (i) resting *P*CO_2_ values (of any water-breather) are typically below its detection limit; (ii) at those *P*CO_2_ values that can be expected in a highly stressed or maximally exercised shark, the measurement error of the i-STAT system is considerable and highly variable (ranging from −5 to −50%); and (iii) complex interactions between *P*O_2_ and *P*CO_2_ measurements make the determination of a single correction factor for i-STAT *P*CO_2_ measurements unreliable, at best.

### Conclusion

Our results indicate that the i-STAT system with the CG4+ cartridge is a useful tool to measure pH in sandbar shark whole blood, but replicate measurements are recommended if accurate mean values are required. Although the i-STAT system underestimated pH by ∼0.1 pH units, a correction for this bias seems possible with the linear equations we provide. In contrast, i-STAT *P*O_2_ measurements on sandbar shark whole blood were associated with a high and variable measurement error, while measurements of *P*CO_2_ were likewise problematic, with resting *in vivo* values being below the detection limit. Therefore, we cannot recommend the i-STAT system for measuring blood gas tensions (and derived parameters) in sandbar shark or, presumably, other fishes.

The limitations imposed by field research can make the accurate measurement of blood gases and pH difficult, if not impossible. With the i-STAT system, researchers have a reliable tool for measuring blood pH in fishes, with an exceptional ease of operation and portability. However, users should carefully evaluate whether the i-STAT system is the most cost-effective means to generate values for pH and any other validated blood parameters not examined in the present study (e.g. lactate), if other (validated) instruments or assays are available.

## Funding

This study was supported by a Natural Sciences and Engineering Research Council (NSERC) of Canada Discovery Grant to C.J.B. and A.P.F. and an NSERC Accelerator Supplement to C.J.B. A.P.F. holds a Canada Research Chair. This is contribution number 3433 from the Virginia Institute of Marine Science, College of William & Mary. Funding for some of the study supplies, including cartridges, was provided by an anonymous donor supporting J.W.M.'s work.
